# Science becomes trustworthy by constantly questioning itself

**DOI:** 10.1371/journal.pbio.3003334

**Published:** 2025-08-01

**Authors:** Brian A. Nosek

**Affiliations:** 1 Center for Open Science, Charlottesville, Virginia, United States of America; 2 University of Virginia, Charlottesville, Virginia, United States of America

## Abstract

What happens when the greatest strengths of science, such as openness, humility, self-criticism and self-correction, are exploited for political gain? This Perspective calls for scientists to affirm the genuine application of those strengths as the source of its trustworthiness.

## Teaser

What happens when the greatest strengths of science—openness, humility, self-criticism, and self-correction—are exploited for political gain? Scientists affirm the genuine application of those strengths as the source of its trustworthiness.

The scientific community is noted for its healthy, sometimes uncomfortable, and unending reckoning with its own limitations. Recent debates about rigor, reproducibility, and the research reward system have led to numerous proposed reforms, including more transparent practices [1], data sharing [2], and preregistration [3], that aim to improve the credibility of scientific claims [4]. Among the scientific community, these reforms have prompted further reflection, investigation, and debate about their effectiveness and potential applicability across research domains.

Such efforts exemplify a core strength of science: its commitment to self-scrutiny. However, when the US Administration issued the May 2025 Executive Order titled ‘Restoring Gold Standard Science’, it suggested that the failure of individual studies to achieve a list of transparency and rigor-related practices is a justification for disqualification of the evidence, and could even potentially be considered misconduct [5,6]. This reframed science’s commitment to identifying limitations as a weakness and has set a standard that is rarely achieved by any individual study. The fall-out from the Executive Order has prompted some difficult questions. Are scientists undermining trust by acknowledging flaws? And how should scientists respond when the tools of accountability are misappropriated [7]?

By its very nature, science is a decentralized enterprise that invites skepticism. No one study can be perfect; trust in science is earned by accumulating evidence with an unrelenting commitment to getting it right [8]. This means welcoming doubt, encouraging dissent and, when required, pursuing better methods in the face of error and failure. It would be disingenuous, dishonest and, ultimately, counterproductive for scientists to knowingly hide their discipline’s imperfections in the name of public reassurance.

Part of the trustworthiness of scientific findings, therefore, comes from this willingness of researchers to ‘put their own house in order’. Practices such as preregistration, data and code sharing, and post-publication peer review were not imposed from the outside, they emerged from within. In some fields, these practices have become normative and even embedded in policies at funders, journals, and institutions. In other fields, researchers are still exploring how such practices might be useful to them. Such reforms are not signs of weakness or admissions of failure; they are expressions of continuous innovation to improve rigor and advance knowledge production. Yet, even as the community works to improve itself, these efforts are being exploited. Researchers, politicians, and others can seize on candid acknowledgments of uncertainty or bias to dismiss the best-available evidence. When that happens, it can feel like scientists are being punished for their transparency and honesty.

Should we then be seeking a middle ground where scientists can continue to be self-critical, but do so cautiously or privately to avoid alarming and arming others? Not at all. To do so would be to misunderstand both the nature of science and the nature of misrepresentation of research. Instead of circumspection, the appropriate response to distortion is clarity. The responsibility of scientists is to state what they believe to be true based on the evidence available to them, transparently and with intellectual humility. This does not mean that scientists will be of one mind. Disunity and discussion are the engines of inquiry, and a decentralized enterprise cannot control its message. With time, the accumulation of evidence, claims, counterclaims, and more evidence, will converge toward the truth, leaving unsupported ideas on the fringe.

Despite disunity, there is a normative expectation within scholarly research that all parties are well-intentioned and truth-seeking. This norm helps to keep debates focused on the ideas and not the people. Of course, not everyone has positive intentions or the objective of advancing knowledge. Scientists can invite productive engagement, model transparency, and maintain norms of accountability that distinguish between disagreement and distortion. And when invitations to engage genuinely are not accepted, or fail verification, scholarly discourse is ill-equipped to resolve the conflict.

But what should scientists do when their work is misrepresented by other scientists, the media, or the state? No single answer exists, but a substantial scholarly literature contains concrete strategies that can help [9,10]. Some mischaracterizations are trivial or unserious and are best ignored. Others, especially when they carry broader implications or visibility, may require a scholarly response, if only to set the record straight. When the distortion comes from the state itself, the challenge is more profound. The tools of science—reason, evidence, and debate—are poorly suited to confront raw political power asserting state-controlled belief systems. In such cases, scientists can lean into their co-existing identity as citizens, drawing on the tools of civic engagement: protest, organizing, and collective resistance. Confronting totalitarianism is not a scientific activity. Science can try to exist separately from politics, but scientists cannot.

Ultimately, there is no communication strategy that can fix this tension. The path forward is not to retreat from transparency but to embrace it. Inviting the public into the process of knowledge building will reveal why it is slow, imperfect, and indispensable. Science is a human endeavor with flaws and biases. Scientists strive toward truth by identifying and correcting those very flaws. Yes, such openness can be exploited. That is a cost of openness. But a world in which scientists soft-pedal or hide self-criticism would be worse. This alternative would give up core tenets that make science trustworthy in a vain attempt to protect against misappropriation. Abandoning science’s greatest strengths in response to acute political pressure is a recipe for long-term peril.

Just as individual scientists can always be working to improve their skills, approach, and character, so too can the research community be working to improve the social systems through which science advances. Equivocation on self-improvement will result in science earning the distrust that it too often receives. We need to continue to critique our research practices, reform our institutions, speak honestly, and, when democratic ideals are threatened, stand up as citizens to defend free inquiry.
